# Nanoformulations of Rilpivirine for Topical Pericoital and Systemic Coitus-Independent Administration Efficiently Prevent HIV Transmission

**DOI:** 10.1371/journal.ppat.1005075

**Published:** 2015-08-13

**Authors:** Martina Kovarova, Olivia D. Council, Abhijit A. Date, Julie M. Long, Tomonori Nochii, Michael Belshan, Annemarie Shibata, Heather Vincent, Caroline E. Baker, William O. Thayer, Guenter Kraus, Sophie Lachaud-Durand, Peter Williams, Christopher J. Destache, J. Victor Garcia

**Affiliations:** 1 Division of Infectious Diseases, Center for AIDS Research, University of North Carolina at Chapel Hill, School of Medicine, Chapel Hill, North Carolina, United States of America; 2 Department of Pharmacy Practice, Creighton University School of Pharmacy and Health Professions, Omaha, Nebraska, United States of America; 3 Janssen Research and Development, Beerse, Belgium; University of Miami, UNITED STATES

## Abstract

Vaginal HIV transmission accounts for the majority of new infections worldwide. Currently, multiple efforts to prevent HIV transmission are based on pre-exposure prophylaxis with various antiretroviral drugs. Here, we describe two novel nanoformulations of the reverse transcriptase inhibitor rilpivirine for pericoital and coitus-independent HIV prevention. Topically applied rilpivirine, encapsulated in PLGA nanoparticles, was delivered in a thermosensitive gel, which becomes solid at body temperature. PLGA nanoparticles with encapsulated rilpivirine coated the reproductive tract and offered significant protection to BLT humanized mice from a vaginal high-dose HIV-1 challenge. A different nanosuspension of crystalline rilpivirine (RPV LA), administered intramuscularly, protected BLT mice from a single vaginal high-dose HIV-1 challenge one week after drug administration. Using transmitted/founder viruses, which were previously shown to establish *de novo* infection in humans, we demonstrated that RPV LA offers significant protection from two consecutive high-dose HIV-1 challenges one and four weeks after drug administration. In this experiment, we also showed that, in certain cases, even in the presence of drug, HIV infection could occur without overt or detectable systemic replication until levels of drug were reduced. We also showed that infection in the presence of drug can result in acquisition of multiple viruses after subsequent exposures. These observations have important implications for the implementation of long-acting antiretroviral formulations for HIV prevention. They provide first evidence that occult infections can occur, despite the presence of sustained levels of antiretroviral drugs. Together, our results demonstrate that topically- or systemically administered rilpivirine offers significant coitus-dependent or coitus-independent protection from HIV infection.

## Author Summary

When taken consistently, PrEP has been shown to reduce the risk of HIV infection by up to 92% in people who are at high risk. However, PrEP is much less effective if it is not taken consistently. To improve adherence to the drug regimen, several new drug delivery systems, that include novel gel formulations and long-acting delivery systems, are being evaluated. In this manuscript, we used BLT humanized mice, an *in vivo* model of vaginal HIV transmission, to evaluate two novel delivery systems for HIV prevention. In the first approach, we combined the highly efficient encapsulation of antiretroviral drugs into nanoparticles with a thermosensitive gel that remains liquid at room temperature and solidifies at body temperature. Our results showed that this delivery system provided significant protection from HIV vaginal infection. In a second approach, we evaluated a long-acting nanoparticle formulation for coitus-independent protection from HIV acquisition. Our results showed that a single injection of the long-acting antiviral drug also resulted in reduced HIV infection. However, protection was not complete and transmission was concealed by a significant delay in the onset of plasma viremia that could result in superinfection by two different viruses administered up to four weeks apart.

## Introduction

Although the annual number of new HIV infections continues to decline, the global HIV-1 pandemic remains an unprecedented public health problem, with 2.1 million new infections in 2013 and an estimated 35 million people already infected [1]. This highlights the urgent need for effective and safe prevention strategies for HIV infection. With the continued absence of an effective vaccine, the efficacy of various antiretrovirals (ARVs) has been evaluated as pre-exposure prophylaxis (PrEP). HIV PrEP refers to the strategy of using ARV drugs to decrease the risk of HIV infection in uninfected individuals who are at high risk of infection. Multiple clinical trials, including the CAPRISA 004, the Chemoprophylaxis for HIV Prevention in Men (iPrEx), the Partners PrEP, and the TDF2 studies have shown that topical or oral pre-exposure administration of ARVs reduces the risk of HIV-1 infection by 39 to 75% [2–5]. Overall efficacy, as well as low rates of protection in some trials, correlates with adherence to the dosing regimen [3]. To improve adherence and PrEP efficacy, several strategies are being considered. These include effective antiretrovirals, easily administered in single topical or systemic dose pericoitally, and long-acting ARV formulations that release drugs over many weeks systemically, requiring infrequent parenteral administration [6–8].

Rilpivirine (RPV, TMC278), a non-nucleoside reverse transcriptase inhibitor (NNRTI), is a diarylpyrimidine derivative that inhibits HIV reverse transcriptase by binding to a hydrophobic pocket near the active site of the enzyme, and consequently preventing transcription of viral RNA. RPV has activity against wild type and many NNRTI-resistant HIV-1 strains [9]. Although RPV has an excellent profile for HIV prevention, there is currently no information regarding the effectiveness of oral RPV for HIV prevention, and no RPV formulations for topical use have been described. Recently, a long-acting crystalline nanoparticle suspension of RPV (RPV LA) has been developed, with the objective of providing drug exposure over extended periods of time following intramuscular administration [10]. A single intramuscular injection of RPV LA provided sustained release of RPV into plasma over 3 months in dogs, 2 months in rats, and 3 weeks in mice [10, 11]. In humans, a single intramuscular administration of RPV LA leads to substantial levels of RPV in plasma, cervico-vaginal fluid and vaginal tissue for 84 days. RPV levels measured at multiple sites of HIV transmission suggest a potential role for RPV LA as coitus-independent PrEP in humans [10, 12].

Animal models are essential to the effective evaluation of new HIV prevention strategies. For example, rhesus macaques (*Macaca mulatta*) and pigtail macaques (*Macaca nemestrina*) were recently used to test whether a long acting formulation of an integrase inhibitor could prevent transmission of HIV *via* rectal or vaginal routes [13–15]. However, the species-specific tropism of HIV prevents the evaluation of relevant viruses, including transmitted/founder viruses, for *in vivo* challenges in these models [16, 17]. Instead, chimeric simian/human immunodeficiency viruses (SHIVs) must be used. Here we tested the efficacy of a topical pericoital and a long-acting systemic nanoformulation of RPV to prevent vaginal HIV-1 transmission using humanized bone marrow/liver/thymus mice (BLT). BLT mice are immunodeficient mice individually bioengineered to express a *de novo*-generated human immune system distributed throughout each animal [18–21], allowing infection with a variety of transmitted/founder HIV-1 isolates *via* relevant routes of transmission. The mouse female reproductive tract (FRT) has anatomic similarities to that of humans, despite its smaller size and presence of two uterine horns that merge to form the main body of uterus. The murine vagina and ectocervix are covered with stratified squamous epithelium, whereas the endocervix and uterus consist of a simple columnar epithelium. The physical barrier that HIV would encounter is, therefore, somewhat similar to that in humans [22]. We previously demonstrated the presence of human CD4+ T cells, macrophages and dendritic cells throughout the mouse female reproductive tract (FRT), that render BLT mice susceptible to vaginal HIV-1 transmission [23, 24]. Both topical and systemic HIV prevention interventions, which parallel human clinical trials, have been successfully performed in BLT mice [23, 25–28]. These studies validate BLT mice as a suitable model for the evaluation of novel or improved drug formulations for the prevention of HIV transmission.

## Results

### Approach for the evaluation of new RPV formulations for the prevention of vaginal HIV infection in humanized BLT mice

**Fig 1:**
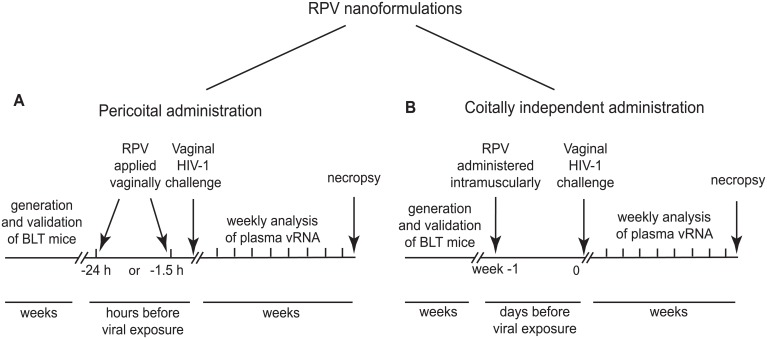
Experimental design for the evaluation of efficacy of RPV nanoformulations in prevention of vaginal HIV transmission in humanized BLT mice. (A) Experimental design for evaluation of efficacy of PLGA nanoparticles loaded with RPV (PLGA/ RPV NPs) in thermosensitive gel as a pericoital PrEP administered as a single topical application 1.5 h or 24 h before HIV-1 challenge. (B) Experimental design for evaluation of efficacy of long acting nanosuspension of RPV administered in a single dose intramuscularly 1 week before HIV-1 challenge, as a coitus-independent approach to prevent vaginal HIV transmission.

We used humanized BLT mice to test two different strategies to prevent vaginal HIV transmission by RPV. Each strategy used a distinct nanotechnology formulation of RPV. RPV gel, a potential pericoital microbicide (developed at Creighton University, Omaha, Nebraska USA), was applied to the vaginal mucosa in single doses 1.5h or 24h before HIV challenge (Fig 1A). In other studies, a coitus-independent, systemic long-acting formulation of rilpivirine RPV LA (developed by Janssen Research and Development, Beerse, Belgium), was administered intramuscularly, 1 week before HIV-1 challenge (Fig 1B). The presence of plasma viral RNA was monitored over time as an early indication of infection. At the end of these experiments, multiple tissues were analyzed for the presence of cell-associated viral DNA. Only animals treated with RPV nanoformulations, and negative for both viral RNA and DNA in peripheral blood and tissues, were considered protected from vaginal HIV-1 transmission.

### RPV, encapsulated in PLGA nanoparticles and delivered in a thermosensitive gel, inhibits HIV-1 infection *in vitro*

**Fig 2:**
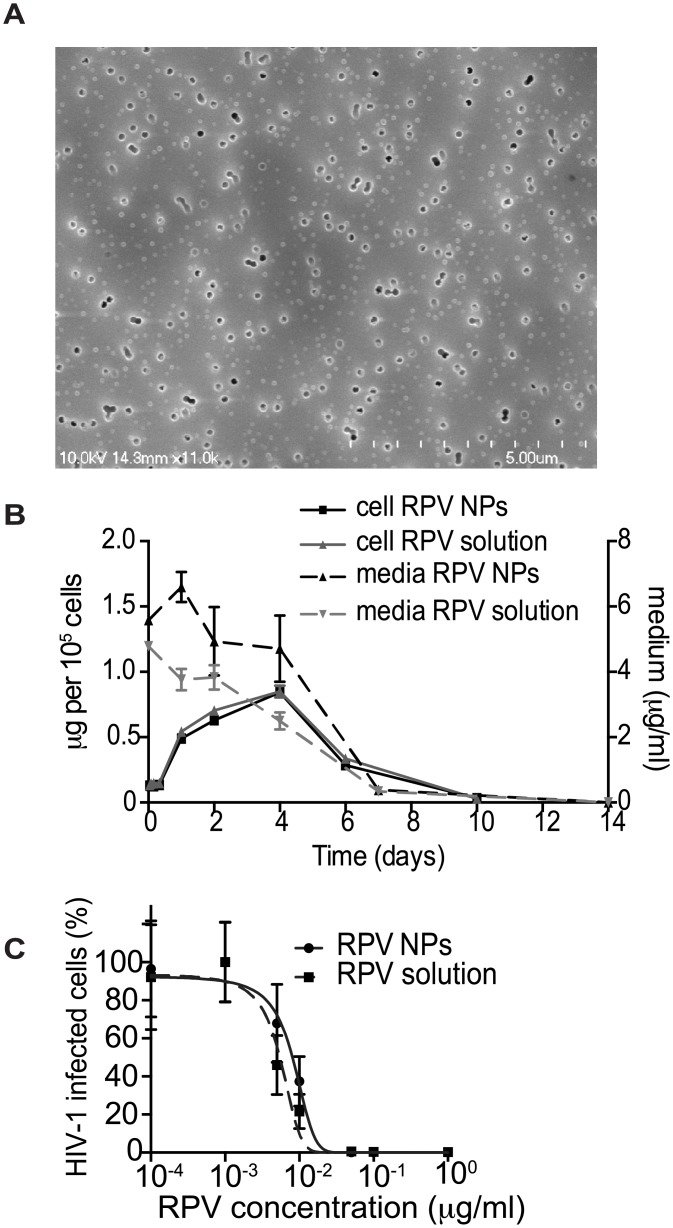
*In vitro* characterization of PLGA/RPV NPs in thermosensitive gel. (A) Scanning electron microscope image of PLGA/RPV nanoparticles. (B) RPV uptake by HeLa cells. Cells were incubated with 5 μg/ml RPV in solution or in the PLGA/RPV NP formulation. Intracellular RPV and RPV in medium were analyzed by HPLC (n = 3). (C) *In vitro* analysis of the inhibition of HIV infection by PLGA/ RPV NPs. TZM-bl HIV indicator cells were treated with the indicated concentrations of RPV solution or PLGA/RPV NPs. Cells were challenged with HIV-1_NLX_ 24 h after RPV treatment. Infection of cells was evaluated by ONE-Glo assay 48 h post infection (n = 3). Data were normalized to luminescence of untreated cells (100%); p = 0.0963.

The majority of clinical trials evaluating pericoital HIV prevention approaches, including CAPRISA 004, used conventional gels for vaginal microbicide delivery. However, such gels have major disadvantages, including gel leakage, uneven distribution, and messiness, which can decrease adherence to the dosing regimen [29]. To overcome these potential drawbacks, we formulated poly(lactic-co-glycolic acid) (PLGA) nanoparticles loaded with RPV (PLGA/RPV NP), in a thermosensitive gel which is liquid at room temperature but highly viscous at body temperature. This property minimizes chances of gel leakage. Moreover, as reported previously, thermosensitive pluronic gels are insensitive to dilution by simulated vaginal fluid [30–33]. PLGA NPs are US FDA-approved biodegradable particles, which can encapsulate ARV and provide their sustained release [34–38]. PLGA/RPV NPs were prepared by emulsion-solvent evaporation. Average particle size was 66.0 ± 4.2nm (mean ± SEM, n = 3) measured by dynamic light scattering, which was further validated using Scanning Electron Microscopy (Fig 2A). The average polydispersity index was 0.14 ± 0.05, zeta potential of the NPs was -10.96 ± 1.4mV (mean ± SEM, n = 3). The RPV encapsulation efficiency in the polymeric nanoparticle, determined by an indirect method as described in the Materials and Methods section, was 98 ± 0.7% and the RPV loading in nanoparticles was ~ 5% w/w of polymer. As shown in Fig 2B, the intracellular uptake of RPV by HeLa cells cultured in presence of a 5μg/ml RPV solution or in the presence of 5μg/ml RPV in PLGA/RPV NPs, was comparable. In TZM-bl indicator cells, PLGA/RPV NPs showed similar *in vitro* inhibition of HIV-1 infection as RPV in solution (Fig 2C). Together, these data confirmed the potential of PLGA/RPV NPs to effectively deliver ARV to target cells. For *in vivo* evaluation in BLT humanized mice, PLGA/RPV NPs were formulated into a previously characterized thermosensitive gel containing Pluronic F127 and Pluronic F68 [32]. The concentration of RPV in thermosensitive gel was 0.876mg/ml.

### Distribution of PLGA/RPV nanoparticles in thermosensitive gel in the female reproductive tract of BLT mice and prevention of vaginal HIV acquisition after vaginal administration

**Fig 3:**
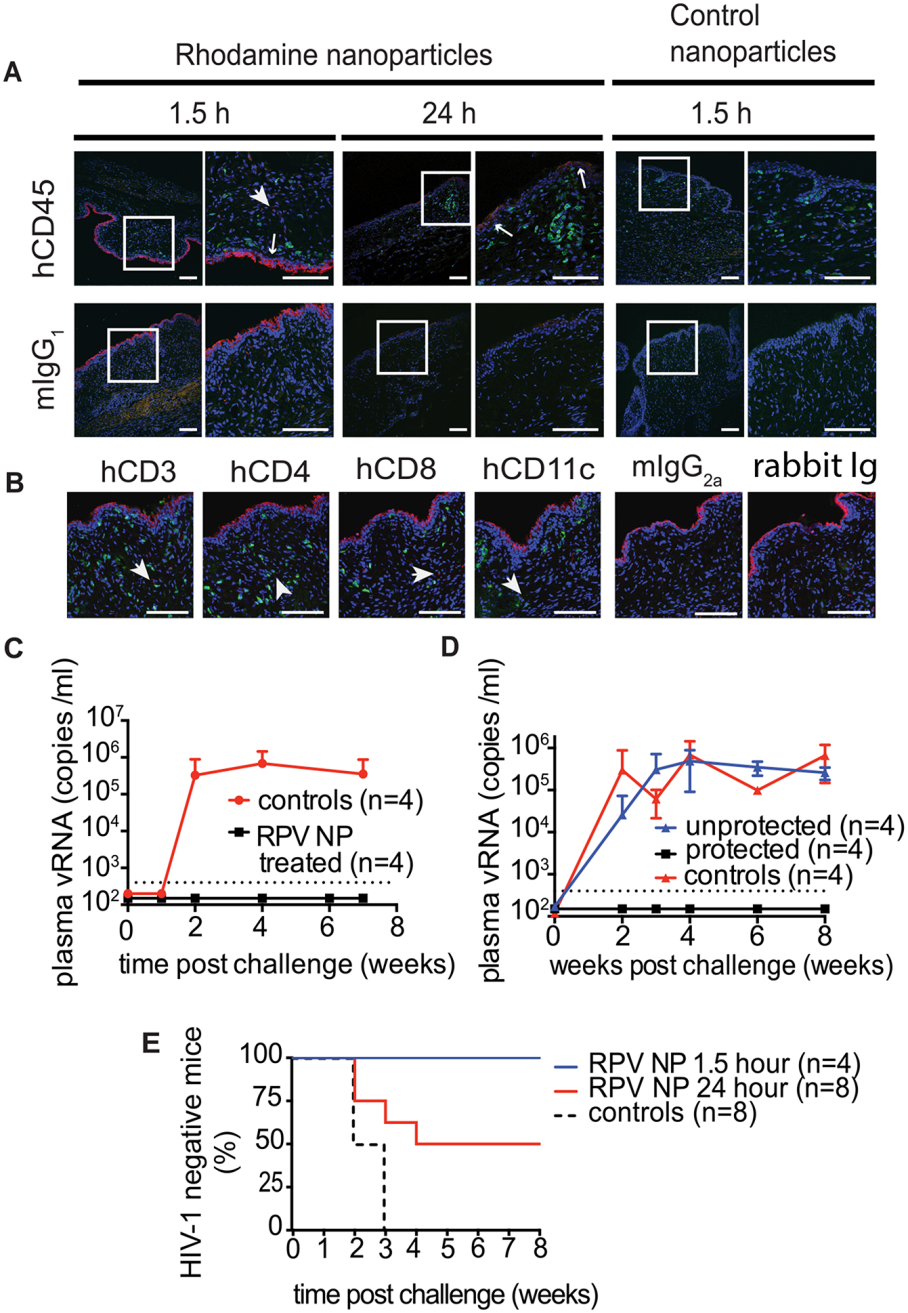
Distribution of rhodamine-labeled NPs in thermosensitive gel in the mouse female reproductive tract (FRT) and the ability of PLGA/RPV to offer pericoital protection against HIV-1 transmission. (A, B) Distribution of rhodamine-labeled PLGA nanoparticles in transverse sections of mouse FRT. Nanoparticles in thermosensitive gel were administered vaginally to humanized BLT mice. FRT was isolated and processed at indicated times and sections were stained for hCD45 (A), hCD3, hCD4, hCD8, hCD11c (B) and DAPI (A, B). Control nanoparticles contain only PLGA. Scale bar = 100 μm. Arrowheads are showing nanoparticles in tissue, arrows are showing nanoparticles on the edge of vaginal epithelium. (C, D) Protection of BLT mice from vaginal HIV-1 infection by topically applied thermosensitive gel containing PLGA/RPV NPs (20 μl of gel, 17.5 μg of RPV per mouse). Controls were treated with vehicle or with thermosensitive gel containing blank NPs. Mice were exposed vaginally to HIV-1_RHPA_ 1.5 h (n = 4) (C) or 24 h (n = 8, two independent experiments) (D) after vaginal administration of gels. Viral RNA was quantified by real time PCR (RT PCR) with a limit of quantitation (LOQ) of 400 copies of RNA per ml (dotted line); graphs represent means ±SD. (E). Kaplan-Meier plots representing the percentage of BLT mice protected by PLGA/RPV NPs in thermosensitive gel over time until the first peripheral blood viral RNA detection. Protected animals were negative for viral RNA in plasma as well as viral DNA in tissue analyzed after necropsy. Statistical analysis: Log-rank (Mantel-Cox) test; controls vs. 1.5 h p = 0.0084, controls vs. 24 h p = 0.0582

In humans, cervicovaginal mucus is a significant barrier and clearance mechanism that limits vaginal drug delivery and retention. To achieve sustained drug release and maintain protective drug concentrations during pre-exposure prophylaxis, drug-loaded nanoparticles need to penetrate cervicovaginal mucus and be efficiently distributed across the female reproductive tract [39]. The ability of PLGA/RPV NPs in thermosensitive gel to distribute in the FRT of humanize mice was evaluated using rhodamine-labeled PLGA NPs. Rhodamine-labeled PLGA NPs in thermosensitive gel were instilled into the mouse vagina and their presence and localization was assessed by confocal microscopy. Ninety minutes after vaginal administration, the fluorescence signal was seen as a continuous layer at the luminal site of the vaginal epithelium. Interestingly, some fluorescence, although with much lower intensity, was still found on the vaginal epithelium 24h after administration. Fluorescence signals were also observed deeper in the tissue in close proximity of hCD45, hCD3, hCD4, hCD8, and hCD11c cells (Fig 3A and 3B). Given the stability of Rhodamine encapsulation in this type of nanoparticles [40], these results demonstrate that PLGA nanoparticles, delivered in thermosensitive gel, can reach the vaginal epithelium and the location of HIV target cells, and persist for 24h.

To test the effectiveness of PLGA/RPV NPs in preventing HIV-1 vaginal transmission, humanized BLT mice were topically treated with PLGA/RPV NPs in thermosensitive gel (20μl of gel, 17.5μg of RPV per mouse; n = 12), thermosensitive gel containing PLGA NP without RPV, or vehicle (n = 8). Treated animals were challenged 1.5h (n = 4) or 24h (n = 8) after gel application with a high dose of HIV-1_RHPA_, a CCR5-tropic transmitted/founder virus (3.1×10^5^ TCID) [16]. The presence of plasma viral RNA in peripheral blood was determined at intervals thereafter. No plasma viral RNA was found in the peripheral blood of any of the animals challenged with HIV-1 1.5h after the administration of PLGA/RPV NPs in thermosensitive gel (0/4) nor in 4/8 of the animals that received PLGA/RPV NPs in thermosensitive gel 24h prior to exposure to HIV-1 (p = 0.0084 and p = 0.0582 respectively). Seven to eight weeks post-exposure, cells isolated from multiple organs were analyzed for the presence of viral DNA. The lack of detectable cell-associated viral DNA in tissues confirmed the absence of HIV infection in these animals (Fig 3C–3E, S1 Table). The presence of plasma viral RNA and cell-associated viral DNA in peripheral blood and tissues of all the control mice confirmed efficient HIV transmission (Fig 3C and 3D). The protection of all BLT mice challenged with HIV-1 1.5h after treatment with PLGA/RPV NPs in thermosensitive gel, and the protection of 50% of the mice treated 24h prior to challenge with HIV-1, demonstrated the effectiveness of PLGA/RPV NPs in HIV prevention.

### Administration of a single dose of RPV LA results in sustained plasma levels of drug

**Fig 4:**
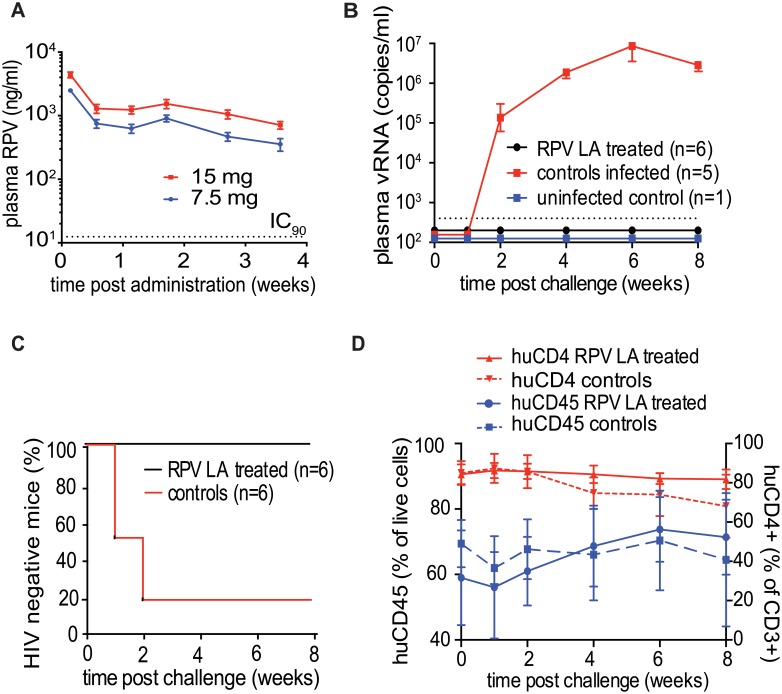
Ability of RPV LA to offer coitus-independent protection from vaginal HIV-1 transmission in BLT mouse. (A) Longitudinal analysis of RPV levels in plasma of NSG mice injected intramuscularly once with 7.5 mg or 15 mg RPV LA (n = 4 for each group, dotted line indicates IC_90_). (B) Plasma viral RNA in BLT mice challenged vaginally with HIV-1_CH040_ (3.5×10^5^ TCID), a transmitted/founder virus 1-week post administration of RPV LA (15 mg, n = 6) or vehicle (n = 6) intramuscularly. Shown are plasma viral RNA (dotted line-LOQ 400 copies of RNA per ml of plasma). (C) Kaplan-Meier plots representing the percentage of BLT mice protected from HIV transmission by RPV LA as a function of the number of weeks post challenge until the first peripheral blood viral RNA detection. Protected animals were negative for viral RNA in plasma and viral DNA in tissue analyzed after necropsy (P = 0.0047, Log rank/Mantel Cox test). (D). Human CD45 cell levels in peripheral blood (percent of total live cells) and human CD4 levels (percent of human CD3 positive cells) were analyzed by flowcytometry at the indicated times. Solid lines: RPV LA treated mice, dashed lines: control animals.

To evaluate the efficacy of a coitus-independent systemic RPV LA formulation to prevent HIV-1 infection, we first determined the plasma RPV concentrations in mice for 28 days after intramuscular injections of either 15mg (50μl of 300mg/ml RPV LA nanosuspension) or 7.5mg (25μl of 300mg/ml RPV LA nanosuspension) of RPV LA (n = 4 per group). As shown in Fig 4A, high levels of RPV were detected in the plasma of all treated animals 24h post-injection. Plasma drug levels decreased rapidly over the following 4 days. After this point, the levels of RPV in plasma remained relatively constant for 10 days and then decreased gradually over the next two weeks. Throughout the 28 days of the study, RPV plasma levels exceeded the protein-adjusted IC_90_ of 12ng/ml [41]. These results demonstrated that a single intramuscular injection of RPV LA resulted in sustained levels of drug in plasma in mice and established its potential to serve as a prevention strategy for intermittent use.

### A single administration of RPV LA efficiently prevents HIV-1 infection in BLT humanized mice

BLT mice (n = 12) were injected intramuscularly with 15mg of RPV LA (n = 6), vehicle or left untreated (n = 6). One week later, mice were challenged vaginally with a high dose (3.5×10^5^ TCID) of HIV-1_CH040_, a transmitted/founder virus [17]. Viral RNA in plasma was evaluated over the following 8 weeks. Five out of six control mice were infected within 2 weeks after challenge, as evidenced by the presence of viral RNA in plasma. In sharp contrast, no viral RNA was detected in the plasma of the animals treated with RPV LA (Fig 4B, S2 Table). Analysis of tissue DNA of RPV LA-treated mice demonstrated the absence of viral DNA in all samples analyzed (S2 Table, Fig 4C). These results demonstrated that a single administration of RPV LA 7 days prior to challenge offered significant protection from vaginal HIV-1_CH040_ infection. It should be noted that longitudinal flow cytometry analysis of the mouse peripheral blood confirmed that the absence of viral RNA and DNA in the mice treated with RPV LA and exposed to HIV-1_CHO40_ was not due to a loss or to reduced levels of human CD45^+^ cells or human CD3^+^CD4^+^ cells throughout the course of the experiment. Only in the infected mice were we able to demonstrate a gradual decrease in the levels of human CD3^+^CD4^+^ cells (Fig 4D).

### Efficacy of RPV LA in preventing HIV infection after two high dose challenges by different HIV isolates 1 and 4 weeks after drug administration

**Fig 5:**
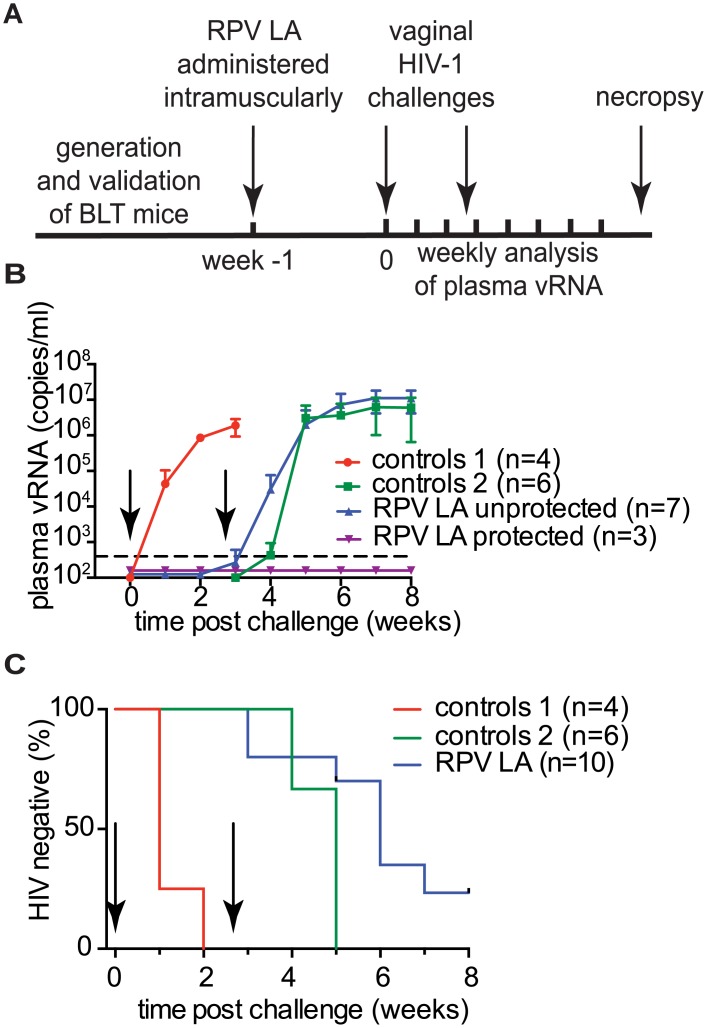
Analysis of RPV LA protection after exposure to high doses of multiple transmitted/founder viruses and an early passage primary isolate. (A) Experimental design. BLT mice were challenged with CH040, RHPA or JR-CSF HIV-1 isolates 1 week after RPV LA administration. Four weeks after RPV LA administration, mice were challenged again, but this time with HIV-1_THRO_, a transmitted/founder virus. (B) Plasma viral load in RPV LA treated BLT mice (n = 10) and controls (n = 4 for 1^st^ challenge, n = 6 for 2^nd^ challenge). Dash line indicates LOQ. Data are presented as mean ± SD. (C) Kaplan-Meier plots representing the percentage of BLT mice protected against HIV transmission by RPV LA intramuscular injection as a function of the number of weeks post 1^st^ and 2^nd^ challenges until the first peripheral blood viral RNA detection. Arrows in panels (B) and (C) indicate time of 1^st^ and 2^nd^ challenges. RHPA (n = 2) and CH040 (n = 2) were used as controls for the first challenge (Control 1); for the 2^nd^ challenge, all control animals were exposed to THRO (n = 6; Control 2); RPV LA indicates RPV LA treatment. Statistical analysis: Log-rank (Mantel-Cox) test, 1^st^ and 2^nd^ challenge were analyzed separately, controls 1 vs. 1^st^ challenge p = <0.0001, controls 2 vs. 2^nd^ challenge p = 0.0038.

The overall experimental approach to evaluate the ability of RPV LA to prevent vaginal HIV transmission after two high dose challenges is shown in Fig 5A. BLT mice received a single 15mg intramuscular injection of RPV LA (n = 10) or vehicle (n = 4). Mice were challenged one week later with a high dose of either HIV-1_JR-CSF_ [an early passage CCR5-tropic primary isolate] (n = 3, TCID 3.5×10^5^), HIV-1_CH040_ (n = 4, TCID 3.5×10^5^) or HIV-1_RHPA_ (n = 3, TCID 3.1×10^5^) transmitted/founder CCR5-tropic viruses. Control mice were challenged with HIV-1_CH040_ or HIV-1_RHPA_ (n = 2 each). Plasma viral RNA was monitored over time. Viral RNA was detected in 4/4 of the control mice. Two weeks post-challenge, no viral RNA was detected in any of the animals that received RPV LA (Fig 5b, S2 Table). Four weeks after RPV LA administration (3 weeks after the first challenge), mice were challenged vaginally with HIV-1_THRO_, a different CCR5-tropic transmitted/founder virus (TCID 3.5×10^5^), in parallel with 6 additional control (no drug) BLT mice (also challenged with HIV-1_THRO_). Mice were monitored for the presence of plasma viral RNA for an additional 5 weeks. Seven of ten RPV LA-treated mice became infected within 4 weeks after the second HIV-1 challenge. In order to identify the virus that resulted in the infection of the RPV LA-treated mice, plasma viral RNA from each infected mouse was sequenced. Sequence analysis revealed that, despite the fact that no viral load was detected in plasma for over 2 weeks after the first challenge, 2 of these mice had actually acquired infection from the first challenge. A third mouse acquired infection after both challenges (dually infected mouse). Sequence analysis of the other 4 mice showed that they were only infected with the second challenge virus (Fig 5B, S1 Fig, S3 Table). No mutations associated with RPV resistance were found in the virus present in any of the RPV LA-treated and infected mice. Analysis of DNA from tissues of the three remaining uninfected mice treated with RPV LA revealed the absence of viral DNA in all tissues analyzed and confirmed their protection from HIV transmission after two high dose challenges (Fig 5C). In summary, during the dual challenge experiment, 7/10 RPV LA-treated animals were protected from the first challenge and 4/9 from the second challenge. These results demonstrate that RPV LA offered significant (>80%; p<0.0001) protection from a high dose of virus administered one week later, and partial (44%; p = 0.0038) protection from a second high dose HIV challenge 4 weeks after drug administration.

## Discussion

There have been numerous attempts to prevent HIV infection with the topical application of microbicides [42]. Topical PrEP is based on the premise that blocking HIV at the site of entry offers the best opportunity to prevent HIV infection and avert systemic toxicity. Topical application of non-specific HIV inhibitors has failed to show protection against HIV infection in several large clinical trials [43]. In contrast, the first clinical trial, evaluating the potential of tenofovir in a vaginal gel formulation (CAPRISA 004), demonstrated significant protection [2]. Subsequent trials, using topically applied tenofovir, were not able to demonstrate protection, most likely due to a lack of product use [44, 45]. Here, we evaluated the efficacy of RPV formulated in PLGA nanoparticles suspended in a thermosensitive gel that remains liquid at room temperature but solidifies at body temperature. We evaluated the distribution of nanoparticles in the vagina of BLT mouse using PLGA nanoparticles with encapsulated rhodamine as a surrogate for rilpivirine. We chose rhodamine because it had been previously shown to be released slowly from the PLGA nanoparticles, with initial burst release seen in the first several hours followed by a more sustained, uniform release [40]. Our results showed that rhodamine-PLGA NPs formed a layer on the lumen of the vaginal epithelium. Some of the particles persisted at the vaginal epithelium 24h post-administration. However, PLGA NPs also infiltrated into the tissue, as fluorescence signal was also found in close proximity to HIV target cells. BLT mice, vaginally treated with PLGA/RPV NPs in the thermosensitive gel, were protected against a high dose challenge with a transmitted/founder virus administered 1.5h later. Protection, diminished (by 50%) when BLT mice were challenged 24h after gel administration. These results demonstrated that topical administration of this novel PLGA/RPV NP thermosensitive gel formulation efficiently prevented vaginal HIV transmission in this animal model.

An alternative to topical PrEP is the systemic administration of antiretrovirals for the prevention of HIV acquisition. The results from multiple clinical trials (iPrEx, TDF2 and Partners PrEP study) demonstrate that systemic PrEP can prevent HIV acquisition *via* rectal and vaginal exposure [3–5]. The results from virtually all clinical trials of HIV prevention using antiretrovirals demonstrate that efficacy depends on product usage. Long-acting injectable formulations of antiretrovirals are one of several approaches that are being considered to enhance adherence to preventive measures.

GSK744 (Cabotegravir) LA is an injectable nanosuspension formulation of an integrase inhibitor that has been shown to be safe and to sustain adequate levels of drug when administered intermittently. Recent results from investigations in non-human primates also demonstrate that GSK744 LA can efficiently prevent infection after repeated low dose viral challenges [13–15]. However, it should be noted that this formulation has demonstrated lower efficacy at preventing infection after a high dose challenge [14].

RPV LA is a long-acting injectable nanosuspension formulation of the NNRTI rilpivirine. Sustained levels of RPV in plasma after single intramuscular injection of RPV LA were reported in dogs, rats and mice, and in plasma, cervico-vaginal fluid and vaginal tissue of humans [10, 12]. However, an assessment of RPV LA in preventing HIV-1 vaginal transmission in a relevant animal model has not yet been reported. Here, we showed that a single administration of RPV LA in BLT mice conferred significant protection against a high dose vaginal challenge with HIV-1 one week after drug administration. Our results also demonstrated that a single administration of RPV LA offered protection, albeit reduced, from a second virus challenge 1 month after drug administration. Importantly, while all our control mice were infected within 2 weeks after challenge, breakthrough infections in treated animals were delayed and occurred 3 weeks after first challenge (4 weeks after drug administration) or 2–4 weeks after second challenge (6–8 weeks after drug administration). These results are consistent with a model in which the initial infection occurs at the site of exposure, but is contained by the presence of sustained levels of drug, preventing systemic replication. Once the levels of drug are unable to efficiently inhibit virus replication, viral spread can occur. Due to faster clearance in mice, a sustained level of RPV in plasma after single injection of RPV LA lasts significantly longer in humans than mice (3 months in humans *vs*. 3 weeks in mice, [10, 11]). Therefore, it is reasonable to anticipate a longer protective effect of RPV LA in humans. In summary, our results in humanized BLT mice highlight the potential of RPV as a candidate for HIV pre-exposure prophylaxis in both a topical coitus-dependent thermosensitive gel formulation as well as in a long acting injectable nanosuspension.

## Materials and Methods

### Preparation and characterization of RPV-loaded PLGA nanoparticles

Resomer 752 H (acid terminated poly-lactic-co-glycolic acid; Avg. Mol. Wt. 15000Da) was purchased from Sigma Chemicals (St. Louis, MO, USA). Rilpivirine (RPV) was purchased from Sequoia Research Ltd. (Pangbourne, UK). Potassium dihydrogen phosphate (HPLC grade), acetonitrile (HPLC grade), dimethyl sulfoxide (DMSO, AR Grade), ethyl acetate (AR grade), citric acid (AR grade) and trisodium citrate (AR grade) were purchased from Fisher Scientific Ltd (NJ, USA). Pluronic F127 and Pluronic F68 (BASF, NJ, USA) were received as gift samples. The ultra-pure water was obtained for all the experiments with the use of PURELAB Ultra system (Elga LLC, IL, USA).

RPV-loaded PLGA nanoparticles were prepared using a previously described emulsion-solvent evaporation method [46]. Briefly, Resomer 752H (200mg) and Pluronic F127 (200mg) were dissolved in 3ml ethyl acetate by heating at 40°C in an incubating shaker bath. RPV (10mg) was dissolved in DMSO (50μl) by heating at 40°C in an incubating shaker bath and then transferred to the Resomer 752H solution to obtain a homogenous organic phase. The organic phase was added drop-wise to 10ml ultrapure water and homogenized using a probe sonicator (UP100H; Hielscher USA, Inc., NJ, USA). The resultant oil-in-water emulsion was stirred for 4h using a magnetic stirrer. Due to the photosensitive nature of RPV, the contents in the beaker were protected from light during the evaporation of ethyl acetate. The mean particle size, polydispersity index and zeta potential of the PLGA/RPV nanoparticles were measured in triplicate using dynamic light scattering at an angle of 90° at 25°C, by a “ZetaPlus” Zeta Potential Analyzer (Brookhaven Instruments Corp, NY, USA). For fabricating rhodamine-6G-labeled fluorescent PLGA nanoparticles, RPV was replaced with rhodamine-6G (1mg) and nanoparticles were formulated as described earlier.

#### SEM imaging of nanoparticles

For SEM imaging, PLGA/RPV NPs (~ 30μl) were transferred onto two-sided conductive tape (PELCO Tabs, 12mm OD, TED PELLA, Inc, Redding, CA) mounted on a aluminum stub. PLGA/RPV NPs were air-dried for >24h and sputter-coated with palladium under an argon gas atmosphere, using a Denton Desk V HP TSC Sputter Coater (Denton Vacuum, LLC-USA, Moorestown, NJ). The coated NPs were examined using a Hitachi S4700 field-emission Scanning Electron Microscope (SEM).

#### Encapsulation efficiency

To determine the amount of RPV encapsulated in nanoparticles, PLGA/RPV nanoparticles (0.4ml) were applied to an Amicon Ultra centrifugal filter (Sigma-Aldrich, MO, USA). The filtrate was obtained by spinning the filter at 14000 rpm for 20min at 4°C in an Eppendorf 5417R centrifuge. The amount of free RPV in the filtrate was measured using a validated reverse-phase HPLC method. The encapsulation efficiency was calculated by the following equation:

$$\%\text{EE }=\;(({\text{W}}_{"\text{initial}"}-{\text{W}}_{"\text{free}"})\;/{\text{W}}_{"\text{initial}"})\text{ X}100$$

where ‘W_initial_’ is the amount of RPV/ml of nanoparticle dispersion and W_free_ is amount of RPV/ml of filtrate obtained by centrifugation of nanoparticles. All experiments were performed in triplicate.

### High-pressure liquid chromatography (HPLC)

A reverse phase-HPLC method was developed and validated for determination of RPV from various matrices derived in the topical gel studies. The HPLC apparatus (Shimadzu Corporation, Columbia, MD) consisted of a pump (LC-20AB), system controller (CBM-20A), degasser unit (DGU-20A), refrigerated auto-sampler (SIL-20AC), a UV-Vis detector (SPD-20A) and a column heater (CTO-20A). Samples were run through a C18 pre-column and a Gemini C_18_ reverse-phase [150mm × 4.5mm (I.D.)] with 5μm particle size packing (Phenomenex, Torrance, CA). The mobile phase consisted of acetonitrile and 25mM KH_2_PO_4_ solution (50:50). For HPLC analysis, the flow rate of the mobile phase was at 0.6ml/min, column oven was set at 35°C, injection volume was 20μl and the analysis was carried out at 290nm. The retention time for the RPV was 12.9min. For standard curve, RPV stock solution (1mg/ml) was prepared in methanol. The stock solution was diluted with acetonitrile to obtain solutions of various concentrations. A standard curve was obtained by injecting 0.025–2μg/ml of RPV. The limit of detection for RPV was 8ng/ml. Intra-day and inter-day variability of the analytical method was <10%.

### Development of a thermosensitive vaginal gel containing RPV NPs

A thermosensitive vaginal gel containing PLGA/RPV-NPs was prepared as described earlier [32]. Briefly, the pH of RPV NPs was adjusted to 4.5 with citric acid and sodium citrate. Glycerol (0.225g) was added to PLGA/RPV NPs (10ml) to adjust the osmolarity of nanoparticles. PLGA/RPV NPs were transferred to a screw-capped bottle and Pluronic F127 (2g) and Pluronic F68 (100mg) were added to PLGA/RPV-NPs with intermittent stirring. The screw-capped bottle containing PLGA/RPV NPs and Pluronics was stored overnight in the refrigerator to dissolve Pluronics. On the next day, the dispersions were gently stirred to obtain a homogenous translucent solution. The solution was observed for signs of nanoparticle aggregation and/or phase separation. Thermosensitivity of the gel was confirmed by incubating the gel in a 37°C water bath. The preparation of thermosensitive gel containing Rhodamine-6G-labeled fluorescent PLGA nanoparticles was carried out in a similar manner.

### Cell culture

Human cervical (HeLa) cells were purchased from the American Type Culture Collection (ATCC, Manassas, VA), TZM-bl cells were procured through the NIH AIDS Research and Reference Reagent Program. HeLa and TZM-bl cells were maintained in complete Dulbecco’s Modified Eagle’s Media ((DMEM, MediaTech Inc., Manassas, VA) supplemented with 10% fetal bovine serum (FBS, Hyclone Inc., Utah), 4mM L-glutamine, 100U/ml penicillin and 100μg/ml streptomycin (MP Biomedical Inc., Solon, OH) and maintained in a logarithmic growth phase. All cells were grown at 37°C and 5% CO_2_.

### *In vitro* HIV-1 inhibition by RPV NPs and RPV solution

TZM-bl HIV indicator cells were seeded in 24-well plates at a density of 2 x 10^5^ per well. After 24h, the cells were treated with different concentrations of PLGA/RPV NPs and RPV solution (concentration range: 10μg/ml to 100pg/ml) for 24h. Cells were washed and cultured for 24h in fresh complete DMEM. Cells were infected with HIV-1 _NL4-3_ virus (25μl) for 4h and incubated for an additional 48h. One-Glo reagent (Promega, Madison, WI), supplemented with Triton X-100 (final concentration 0.01%) was added to inactivate virus and to allow for the measurement of luciferase activity. Results were normalized to the luciferase activity of cells infected with virus incubated with plain RPMI medium.

### RPV LA nanosuspension and RPV plasma level analysis

RPV LA was prepared as previously described [11]. Briefly, a sterile isotonic nanosuspension, consisting of rilpivirine particles, was prepared by wet nanomilling of the rilpivirine base, surfactant, and buffer to ensure neutral pH under aseptic conditions. The median particle size was 200nm. Poloxamer 338 (Pluronics F108), a hydrophilic, nonionic surfactant, was used to enhance solubility and stabilize the colloidal suspension against aggregation. The final drug concentration was 300mg/ml.

Plasma was isolated from 0.05–0.1ml peripheral blood samples on EDTA collected from mouse retro-orbital venous sinus and stored at -80°C until analysis. Plasma samples were analyzed individually for unchanged rilpivirine by liquid chromatography-tandem mass spectrometry (LC/MS-MS) as described previously [11].

### Generation of humanized BLT mice

BLT mice were generated as described previously [19, 23, 26, 27, 47, 48]. Briefly, a 1–2mm piece of human fetal liver tissue was sandwiched between two pieces of autologous fetal thymus tissue (Advanced Bioscience Resources, Alameda, CA) under the kidney capsule of sublethally irradiated (0.250Sv) 6–8 wk old NOD.Cg- Prkdcscid Il2rgtm1Wjl/SzJ mice (NSG; The Jackson Laboratory, Bar Harbor, ME). Following implantation, mice were transplanted intravenously with hematopoietic CD34+ stem cells isolated from autologous human fetal liver tissue. Human immune cell reconstitution was monitored by flow cytometrical analysis of the peripheral blood every 2 weeks, as previously described [23, 26, 27, 48]. At the end of experiments, mice were euthanized by exposure to avertin followed by euthanasia. Mice were maintained at the Division of Laboratory Animal Medicine, University of North Carolina at Chapel Hill (UNC-CH).

### Ethics statement

All animal experiments were carried out in accordance with the recommendations in the Guide for the Care and Use of Laboratory Animals of the National Institutes of Health. The protocol was approved by the Institutional Animal Care and Use Committee guidelines of the University of North Carolina (protocol number:12–170).

### Distribution of nanoparticles in the female reproductive tract

Female humanized-BLT mice were anesthetized with Nembutal. Rhodamine-labeled NPs or control nanoparticles (without rhodamine) in thermosensitive gel (20μl) was instilled into the mouse vagina. 1.5h or 24h later, mice were sacrificed and the FRT harvested, fixed in 4% paraformaldehyde solution (SafeFix, Fisher Science) and embedded in OCT compound (Sakura). Mouse vaginal frozen sections (5μm) were stained with monoclonal antibody for hCD45 (Dako, mouse IgG1), hCD3 (Thermo Scientific, rabbit IgG), hCD4 (GenWay, Rabbit IgG), hCD8 (Dako, Mouse IgG1), hCD11c (Leica, mouse IgG2a), mouse IgG1 (Dako), mouse IgG2a (Dako) or rabbit Ig (Dako) after blocking with Background Sniper (Biocare Medical). The sections were then stained with either DyLight 488-conjugated donkey anti-mouse IgG or DyLight 488-conjugated donkey anti-rabbit IgG (Jackson Immunoresearch). All sections were finally counterstained with DAPI (Sigma) and analyzed by confocal microscopy (TCS SP2, Leica).

### Treatment and intravaginal exposure of BLT mice to transmitted/founder HIV-1

For topical administration of PLGA/RPV NP, BLT mice were administered intravaginally with 17.5μg RPV in the form of PLGA/RPV NPs in 20μl thermosensitive gel containing PLGA/RPV NPs. 1.5h or 24h later, the animals were anesthetized with Nembutal and challenged with 4.5×10^5^ TCID HIV transmission/founder virus HIV_RHPA_. Control BLT mice (n = 4) received vehicle or thermosensitive gel with blank nanoparticles, and were challenged with the same transmission/founder virus.

For systemic administration of RPV LA, female BLT mice received single injection of 15 mg of nanosuspension intramuscularly. One week later, mice were anesthetized with Nembutal and intravaginally challenged with transmission/founder viruses (HIV_CHO40_ 3.0×10^5^ TCID or HIV_RHPA_ 4.5×10^5^ TCID) or HIV_JR-CSF_ (7.0×10^5^ TCID). 3 weeks later, uninfected mice were challenged vaginally with transmission/founder HIV_THRO_ (4.0×10^5^ TCID).

Viral stocks were generated by transfecting proviral DNA into 293T cells using Lipofectamine 2000 (Invitrogen) and tissue culture infectious units (TCID) were determined using TZM-bl cells, essentially as we have previously reported [49, 50]. HIV-1 JR-CSF, CHO40, THRO and RHPA were obtained from Dr. Irving Chen and John Kappes via the AIDS Research and Reagent Repository Program.

### Analysis of HIV-1 infection in humanized BLT mice

Infection of BLT mice with HIV-1 was monitored in peripheral blood by determining levels of viral RNA in plasma by one-step real-time reverse transcriptase PCR assay, using the following primers: CATGTTTTCAGCATTATCAGAAGGA, TGCTTGATGTCCCCCCACT, and the MGB-probe carboxyfluorescein (FAM)-CCACCCCACAAGATTTAAACACCATGCTAA-Q (nonfluorescent quencher) (Applied Biosystems) (sensitivity of 400 HIV RNA copies/ml). The percentage of human CD4+ T cells in peripheral blood of BLT mice before challenge (0–2 weeks prior to exposure) and after challenge was determined by flow cytometry with respective antibodies: hCD45-APC, hCD3-FITC, hCD4-PE and hCD8-PerCP (eBioscience). Flow cytometry data were collected using a BD FACSCanto cytometer and analyzed using BD FACSDiva software. The presence of viral DNA in tissues and peripheral blood collected from BLT mice was determined by real-time PCR analysis of DNA extracted from 5×10^4^–4×10^6^ cells from harvested tissue (spleen, lymph nodes, bone marrow, liver, lung, female reproductive tract) or from 15–50μl peripheral blood cells, as previously described [23, 26, 27, 51]; (assay sensitivity of 10 DNA copies per sample).

### Identification of transmitted viruses

Viruses replicating in infected animals were identified by sequence analysis. Viral RNA was isolated from plasma using QIAamp viral RNA columns (Qiagen) according to the manufacturer’s protocol, and cDNA was generated using Superscript III Reverse Transcriptase (Invitrogen) with the primer GTGGGTACACAGGCATGTGTGG. cDNA was amplified by nested PCR using the Expand High Fidelity PCR System (Roche). PCR primers were designed to anneal in regions with the fewest possible primer mismatches to HIV_JR-CSF_, HIV_CH040_, HIV_RHPA_ and HIV_THRO_ sequences. Primer sequences were as follows: outer forward primer, TGCATATTGTGAGTCTGTTACTATGTTTACT; reverse prime CAGGAGCAGATGATACAG; inner forward primer, GTAGGACCTACACCTGTCAAC; reverse primer CCTGCAAAGCTAGGTGAATTGC. Amplified viral DNA was sequenced and compared to sequences of transmitted/founder viruses.

### Statistical analysis

Drug concentrations in plasma over time were compared using Tukey’s multiple comparison test. Statistical differences between treated and control animals in the efficacy of tested nanoformulations in protection protecting from vaginal HIV-1 transmission were determined by log-rank/ Mantel–Cox test. All statistical analyses were performed using GraphPad Prism software (version 6).

## Supporting Information

S1 Fig Plasma viral RNA of individual RPV LA treated animals. (A) and controls for 1^st^ challenge (dotted lines) and 2^nd^ challenge (solid lines)(B) for the experiment depicted in Fig 5. Arrows indicate time of challenges. Experimental details for individual mouse are indicated in S3 Table. RNA was quantified by RT PCR with LOQ 400 copies RNA per ml (dash line).
(EPS)

S1 Table Protection of BLT mice, topically treated with RPV NP in thermosensitive gel, from vaginal HIV-1 transmission. BLT mice with indicated levels of human CD45^+^ (hCD45) cells and human CD3^+^CD4^+^ (hCD4) in peripheral blood were vaginally treated with RPV NP, blank NP or vehicle. At indicated time after the treatment mice were challenged with HIV-1_RHPA_. Presence of viral RNA in plasma was monitored over time. Cell-associated DNA was analyzed in indicated tissues after necropsy. n.a. not analyzed;—negative for viral RNA or DNA; + positive for viral RNA or DNA; org. thymic organoid, FRT female reproductive tract. *mouse #3 was found dead in the cage; analysis of tissue for cell-associated DNA was not possible.
(DOCX)

S2 Table Protection of BLT mice treated with RPV LA from single high dose vaginal challenge with HIV-1. BLT mice with indicated levels of human CD45^+^ (hCD45) cells and human CD3^+^CD4^+^ (hCD4) in peripheral blood were treated intramuscularly with RPV LA formulation (RPV LA) or vehicle. One week after the treatment, mice were challenged with HIV_CH040_. Presence of viral RNA in plasma was monitored weekly. Cell-associated DNA was analyzed in indicated tissue. n.a.: not analyzed;—negative for viral DNA; + positive for viral DNA; org. thymic organoid.
(DOCX)

S3 Table Protection of BLT mice treated with RPV LA from two high dose vaginal challenges with HIV-1. BLT mice with indicated levels of human CD45^+^ (hCD45) cells and human CD3^+^CD4^+^ (hCD4) in peripheral blood were treated intramuscularly with RPV LA formulation (RPV LA) or vehicle. One week after the treatment, mice were challenge with indicated HIV isolates (virus for 1^st^ challenge). Three weeks later 9 mice were challenge with HIV_THRO_ (virus for 2^nd^ challenge). Presence of viral RNA in plasma was monitored weekly. *Viruses in infected mice were identified by sequencing. Cell-associated DNA was analyzed in indicated tissue. n.a.: not analyzed;—negative for viral DNA; + positive for viral DNA; org. thymic organoid. Notes: mouse # 2R1 died while the 2^nd^ inoculation was being administered, mouse #2R4 died 3 weeks after 2^nd^ inoculation; mouse #2R5 died 2 weeks after 2^nd^ inoculation.
(DOCX)
