# Correction: Nanoformulations of Rilpivirine for Topical Pericoital and Systemic Coitus-Independent Administration Efficiently Prevent HIV Transmission

**DOI:** 10.1371/journal.ppat.1005170

**Published:** 2015-10-16

**Authors:** Martina Kovarova, Olivia D. Council, Abhijit A. Date, Julie M. Long, Tomonori Nochi, Michael Belshan, Annemarie Shibata, Heather Vincent, Caroline E. Baker, William O. Thayer, Guenter Kraus, Sophie Lachaud-Durand, Peter Williams, Christopher J. Destache, J. Victor Garcia

The fifth author's name is spelled incorrectly. The correct name is: Tomonori Nochi.
