# Supplementary material for: Nanoformulations of Rilpivirine for Topical Pericoital and Systemic Coitus-Independent Administration Efficiently Prevent HIV Transmission
Source: PLoS Pathog. 2015 Aug 13;11(8):e1005075. doi: 10.1371/journal.ppat.1005075 (PMC4536200; doi:10.1371/journal.ppat.1005075)
Supplement: S1 Table — BLT mice with indicated levels of human CD45+ (hCD45) cells and human CD3+CD4+ (hCD4) in peripheral blood were vaginally treated with RPV NP, blank NP or vehicle. At indicated time after the treatment mice were challenged with HIV-1RHPA. Presence of viral RNA in plasma was monitored over time. Cell-associated DNA was analyzed in indicated tissues after necropsy. n.a. not analyzed;—negative for viral RNA or DNA; + positive for viral RNA or DNA; org. thymic organoid, FRT female reproductive tract. *mouse #3 was found dead in the cage; analysis of tissue for cell-associated DNA was not possible. (DOCX) [file ppat.1005075.s002.docx]

**Table S1:Protection of BLT mice, topically treated with RPV NP in thermosensitive gel, from vaginal HIV-1 transmission**

| Mouse number | | hCD45 (%) | hCD4 (%) | Treatment | Time between treatment and HIV challenge (h) | Viral RNA in peripheral blood | | Presence of viral DNA in tissues | | | | | | | | | |
| --- | --- | --- | --- | --- | --- | --- | --- | --- | --- | --- | --- | --- | --- | --- | --- | --- | --- |
|  |  |  |  |  |  |  |  | peripheral blood | FRT | lymph nodes | spleen | liver | | lung | | bone marrow | org. |
| 1 | | 91 | 93 | Blank NP | 1.5 | + | | + | + | + | + | + | | + | | + | + |
| 2 | | 91 | 93 | Blank NP | 1.5 | + | | + | + | + | + | + | | + | | + | + |
| 3 | | 53 | 89 | vehicle | 1.5* | + | | n.a. | n.a. | n.a. | n.a. | n.a. | | n.a. | | n.a. | n.a. |
| 4 | | 83 | 74 | vehicle | 1.5 | + | | + | + | + | + | + | | + | | + | + |
| 5 | | 92 | 93 | RPV NP | 1.5 | - | | - | - | - | - | - | | - | | - | - |
| 6 | | 90 | 93 | RPV NP | 1.5 | - | | - | - | - | - | - | | - | | - | - |
| 7 | | 90 | 92 | RPV NP | 1.5 | - | | - | - | - | - | - | | - | | - | - |
| 8 | | 87 | 92 | RPV NP | 1.5 | - | | - | - | - | - | - | | - | | - | - |
|  | |  |  |  |  |  | |  |  |  |  |  | |  | |  |  |
| 9 | | 63 | 85 | vehicle | 24 | + | | + | + | + | + | + | | + | | + | + |
| 10 | | 72 | 84 | vehicle | 24 | + | | + | + | + | + | + | | + | | + | + |
| 11 | | 65 | 90 | vehicle | 24 | + | | + | + | + | + | + | | + | | + | + |
| 12 | | 76 | 81 | vehicle | 24 | + | | + | + | + | + | + | | + | | + | + |
| 13 | | 59 | 88 | RPV NP | 24 | - | | - | - | - | - | - | | - | | - | - |
| 14 | 59 | 88 | RPV NP | 24 | - | - | | - | - | - | - | - | | - | - |  |  |
| 15 | 55 | 85 | RPV NP | 24 | + | + | | + | + | + | + | + | | + | + |  |  |
| 16 | 77 | 85 | RPV NP | 24 | + | + | | + | + | + | + | + | | + | + |  |  |
| 17 | 57 | 87 | RPV NP | 24 | - | - | | - | - | - | - | - | | - | - |  |  |
| 18 | 70 | 87 | RPV NP | 24 | + | + | | + | + | + | + | + | | + | + |  |  |
| 19 | 79 | 85 | RPV NP | 24 | + | + | | + | + | + | + | + | | + | + |  |  |
| 20 | 71 | 89 | RPV NP | 24 | - | - | | - | - | - | - | - | | - | - |  |  |

BLT mice with indicated levels of human CD45^+^ (hCD45) cells and human CD3^+^CD4^+^ (hCD4) in peripheral blood were vaginally treated with RPV NP, blank NP or vehicle. At indicated time after the treatment mice were challenged with HIV-1_RHPA_. Presence of viral RNA in plasma was monitored over time. Cell-associated DNA was analyzed in indicated tissues after necropsy. n.a. not analyzed; - negative for viral RNA or DNA; + positive for viral RNA or DNA; org. thymic organoid, FRT female reproductive tract. *mouse #3 was found dead in the cage; analysis of tissue for cell-associated DNA was not possible.
