# Supplementary material for: Nanoformulations of Rilpivirine for Topical Pericoital and Systemic Coitus-Independent Administration Efficiently Prevent HIV Transmission
Source: PLoS Pathog. 2015 Aug 13;11(8):e1005075. doi: 10.1371/journal.ppat.1005075 (PMC4536200; doi:10.1371/journal.ppat.1005075)
Supplement: S2 Table — BLT mice with indicated levels of human CD45+ (hCD45) cells and human CD3+CD4+ (hCD4) in peripheral blood were treated intramuscularly with RPV LA formulation (RPV LA) or vehicle. One week after the treatment, mice were challenged with HIVCH040. Presence of viral RNA in plasma was monitored weekly. Cell-associated DNA was analyzed in indicated tissue. n.a.: not analyzed;—negative for viral DNA; + positive for viral DNA; org. thymic organoid. (DOCX) [file ppat.1005075.s003.docx]

**Table S2:** **Protection of BLT mice treated with RPV LA from single high dose vaginal challenge with HIV-1.**

| Mouse code | hCD45(%) | hCD4(%) | Treatment | Virus used for challenge | Time to viral RNA detection in plasma (weeks) | Presence of viral DNA in tissues | | | | | |
| --- | --- | --- | --- | --- | --- | --- | --- | --- | --- | --- | --- |
|  |  |  |  |  |  | lymph nodes | spleen | liver | lung | bone marrow | org. |
| C2 | 75.4 | 86.7 | vehicle | CH040 | 2 | + | + | + | + | + | + |
| C3 | 61.4 | 78.3 | vehicle | CH040 | 2 | + | + | + | + | + | + |
| C1 | 71.4 | 90.0 | vehicle | CH040 | not infected | n.a. | n.a. | n.a. | n.a. | n.a. | n.a. |
| C4 | 71.2 | 77.5 | none | CH040 | 2 | n.a. | n.a. | n.a. | n.a. | n.a. | n.a. |
| C5 | 82.1 | 75.8 | none | CH040 | 2 | n.a. | n.a. | n.a. | n.a. | n.a. | n.a. |
| C6 | 71.8 | 88.9 | none | CH040 | 2 | n.a. | n.a. | n.a. | n.a. | n.a. | n.a. |
| 1R1 | 80.2 | 88.3 | RPV LA | CH040 | protected | - | - | - | - | - | - |
| 1R2 | 73.2 | 86.8 | RPV LA | CH040 | protected | - | - | - | - | - | - |
| 1R3 | 43.8 | 83.1 | RPV LA | CH040 | protected | - | - | - | - | - | - |
| 1R4 | 48.0 | 78.1 | RPV LA | CH040 | protected | - | - | - | - | - | - |
| 1R5 | 55.9 | 79.1 | RPV LA | CH040 | protected | - | - | - | - | - | - |
| 1R6 | 52.9 | 90.6 | RPV LA | CH040 | protected | - | - | - | - | - | - |

BLT mice with indicated levels of human CD45^+^ (hCD45) cells and human CD3^+^CD4^+^ (hCD4) in peripheral blood were treated intramuscularly with RPV LA formulation (RPV LA) or vehicle. One week after the treatment, mice were challenged with HIV_CH040_. Presence of viral RNA in plasma was monitored weekly. Cell-associated DNA was analyzed in indicated tissue. n.a.: not analyzed; - negative for viral DNA; + positive for viral DNA; org. thymic organoid.
