# Supplementary material for: Nanoformulations of Rilpivirine for Topical Pericoital and Systemic Coitus-Independent Administration Efficiently Prevent HIV Transmission
Source: PLoS Pathog. 2015 Aug 13;11(8):e1005075. doi: 10.1371/journal.ppat.1005075 (PMC4536200; doi:10.1371/journal.ppat.1005075)
Supplement: S3 Table — BLT mice with indicated levels of human CD45+ (hCD45) cells and human CD3+CD4+ (hCD4) in peripheral blood were treated intramuscularly with RPV LA formulation (RPV LA) or vehicle. One week after the treatment, mice were challenge with indicated HIV isolates (virus for 1st challenge). Three weeks later 9 mice were challenge with HIVTHRO (virus for 2nd challenge). Presence of viral RNA in plasma was monitored weekly. *Viruses in infected mice were identified by sequencing. Cell-associated DNA was analyzed in indicated tissue. n.a.: not analyzed;—negative for viral DNA; + positive for viral DNA; org. thymic organoid. Notes: mouse # 2R1 died while the 2nd inoculation was being administered, mouse #2R4 died 3 weeks after 2nd inoculation; mouse #2R5 died 2 weeks after 2nd inoculation. (DOCX) [file ppat.1005075.s004.docx]

**Table S3: Protection of BLT mice treated with RPV LA from two high dose vaginal challenges with HIV-1.**

| Mouse code | hCD45(%) | hCD4(%) | Treatment | Virus for 1^st^ challenge | Virus for 2^nd^ challenge | Infecting virus | Time to virus detection in plasma (weeks) | Presence of viral DNA in tissues | | | | | |
| --- | --- | --- | --- | --- | --- | --- | --- | --- | --- | --- | --- | --- | --- |
|  |  |  |  |  |  |  |  | lymph nodes | spleen | liver | lung | bone marrow | org. |
| 2C1 | 61.9 | 83.6 | vehicle | None | THRO | THRO | 2 | + | + | + | + | + | + |
| 2C2 | 59.4 | 78.6 | vehicle | None | THRO | THRO | 1 | + | - | + | + | + | + |
| 2C3 | 75 | 79 | vehicle | None | THRO | THRO | 2 | + | - | + | + | + | + |
| 2C4 | 71.2 | 78.6 | vehicle | None | THRO | THRO | 2 | + | + | + | + | + | + |
| 2C5 | 86.5 | 74.2 | none | None | THRO | THRO | 2 | n.a. | n.a. | n.a. | n.a. | n.a. | n.a. |
| 2C6 | 73.4 | 76.1 | none | None | THRO | THRO | 1 | n.a. | n.a. | n.a. | n.a. | n.a. | n.a. |
| 1C1 | 85 | 75.8 | vehicle | CH040 | None | CH040 | 1 | n.a. | n.a. | n.a. | n.a. | n.a. | n.a. |
| 1C2 | 81.8 | 81.7 | vehicle | CH040 | None | CH040 | 1 | n.a. | n.a. | n.a. | n.a. | n.a. | n.a. |
| 1C3 | 69.8 | 78.5 | none | RHPA | None | RHPA | 1 | n.a. | n.a. | n.a. | n.a. | n.a. | n.a. |
| 1C4 | 86.8 | 72.8 | none | RHPA | None | RHPA | 2 | n.a. | n.a. | n.a. | n.a. | n.a. | n.a. |
| 2R1 | 85.1 | 82.4 | RPV LA | CH040 | THRO | CH040 | 3 | + | + | + | + | + | + |
| 2R2 | 84 | 85.2 | RPV LA | CH040 | THRO | THRO* | 3 | + | + | + | + | + | + |
| 2R3 | 89.8 | 83.2 | RPV LA | CH040 | THRO | THRO* | 3 | + | + | + | + | + | + |
| 2R4 | 56.3 | 87.9 | RPV LA | CH040 | THRO | None | protected | - | - | - | - | - | - |
| 2R5 | 66.4 | 87.6 | RPV LA | RHPA | THRO | None | protected | - | - | - | - | - | - |
| 2R6 | 75.3 | 88.5 | RPV LA | RHPA | THRO | RHPA* THRO* | 4 2 | + | + | + | + | + | + |
| 2R7 | 75.8 | 83.3 | RPV LA | RHPA | THRO | None | protected | - | - | - | - | - | - |
| 2R8 | 85.2 | 83 | RPV LA | JR-CSF | THRO | THRO* | 4 | + | + | + | + | + | + |
| 2R9 | 80.5 | 80.8 | RPV LA | JR-CSF | THRO | THRO* | 2 | + | + | + | + | + | + |
| 2R10 | 84.9 | 84 | RPV LA | JR-CSF | THRO | JR-CSF* | 3 | + | + | + | + | + | + |

BLT mice with indicated levels of human CD45^+^ (hCD45) cells and human CD3^+^CD4^+^ (hCD4) in peripheral blood were treated intramuscularly with RPV LA formulation (RPV LA) or vehicle. One week after the treatment, mice were challenge with indicated HIV isolates (virus for 1^st^ challenge). Three weeks later 9 mice were challenge with HIV_THRO_ (virus for 2^nd^ challenge). Presence of viral RNA in plasma was monitored weekly. *Viruses in infected mice were identified by sequencing. Cell-associated DNA was analyzed in indicated tissue. n.a.: not analyzed; - negative for viral DNA; + positive for viral DNA; org. thymic organoid. Notes: mouse # 2R1 died while the 2^nd^ inoculation was being administered, mouse #2R4 died 3 weeks after 2^nd^ inoculation; mouse #2R5 died 2 weeks after 2^nd^ inoculation.
